# Intercerebral autoregulation index consistency in the derivation of CPPopt, MAPopt, and BISopt in humans: A scoping review

**DOI:** 10.14814/phy2.70660

**Published:** 2025-12-19

**Authors:** Rakibul Hasan, Karl Zhang, Kevin Y. Stein, Angela Buchel, Tobias Bergmann, Amanjyot Singh Sainbhi, Nuray Vakitbilir, Isuru Herath, Noah Silvaggio, Mansoor Hayat, Jaewoong Moon, Frederick A. Zeiler

**Affiliations:** ^1^ Department of Biomedical Engineering, Price Faculty of Engineering University of Manitoba Winnipeg Manitoba Canada; ^2^ Undergraduate Genetics Program, Faculty of Science University of Manitoba Winnipeg Manitoba Canada; ^3^ Undergraduate Medical Education, Rady Faculty of Health Sciences University of Manitoba Winnipeg Manitoba Canada; ^4^ Department of Human Anatomy and Cell Science, Rady Faculty of Health Sciences University of Manitoba Winnipeg Manitoba Canada; ^5^ Section of Neurosurgery, Department of Surgery, Rady Faculty of Health Sciences University of Manitoba Winnipeg Manitoba Canada; ^6^ Department of Clinical Neuroscience Karolinska Institutet Stockholm Sweden; ^7^ Pan Am Clinic Foundation Winnipeg Manitoba Canada

**Keywords:** autoregulatory indices, BISopt, cerebrovascular reactivity, CPPopt, MAPopt

## Abstract

The optimization of cerebral perfusion and sedation using autoregulation‐derived physiologic targets such as optimal cerebral perfusion pressure (CPPopt), optimal mean arterial pressure (MAPopt), and optimal bispectral index (BISopt) has emerged as a promising strategy in neurocritical and perioperative care. However, the reliability and comparability of these optimal (Opt) parameters across different autoregulatory indices remain uncertain. This paper systematically reviews and synthesizes literature comparing CPPopt, MAPopt, and BISopt derived from invasive and noninvasive indices. Following PRISMA‐ScR guidelines, studies directly comparing CPPopt, MAPopt, or BISopt from at least two indices were included. Ten studies compared CPPopt, mostly in traumatic brain injury, with mean values between 70 and 76 mmHg. Nine studies compared MAPopt, reporting strong correlations between transcranial doppler‐, near‐infrared spectroscopy‐, and intracranial pressure‐derived indices across populations, though limits of agreement were wide. One study compared BISopt across indices, showing internal consistency, while two cross‐Opt studies found little correlation between BISopt and CPPopt or MAPopt. CPPopt and MAPopt appear physiologically robust across indices, supporting translational potential in both invasive and noninvasive settings. BISopt may represent a distinct optimization domain related to sedation rather than perfusion. Methodological heterogeneity and limited outcome validation remain barriers. Future work should emphasize standardization, multimodal integration, and outcome‐driven trials.

## 1 INTRODUCTION

Cerebrovascular autoregulation (CA) is a fundamental physiological mechanism that maintains stable cerebral blood flow (CBF) despite fluctuations in cerebral perfusion pressure (CPP) (Van Den Dool et al., 2023). This process ensures adequate oxygen and nutrient delivery to brain tissue across varying systemic pressures (Fantini et al., 2016). One way to assess the integrity of CA is through cerebrovascular reactivity (CVR), which quantifies the brain's ability to dynamically adjust vascular tone in response to changes in perfusion or metabolic demand (Duffin et al., 2018). When autoregulation is impaired, as commonly observed in conditions such as traumatic brain injury (TBI), stroke, anesthesia, or critical illness, the brain becomes vulnerable to secondary ischemic or hyperemic injury (Lassen, 1959; Toth et al., 2016). Over the past two decades, advances in multimodal neuromonitoring have enabled continuous bedside assessment of CA and CVR, allowing the development of individualized physiologic targets derived from these mechanisms (Casault et al., 2022; Vitt et al., 2023).

Among these targets, optimal cerebral perfusion pressure (CPPopt), derived primarily from the pressure reactivity index (PRx) and related indices, has been widely studied in patients with traumatic brain injury (TBI) (Steiner et al., 2002; Aries et al., 2012; Depreitere et al., 2014). CPPopt represents the perfusion pressure at which cerebrovascular reactivity is most intact and has been associated with improved outcomes when maintained within individualized ranges (Bögli, Olakorede, Beqiri, Chen, et al., 2025; Liu et al., 2017). Parallel to this, optimal mean arterial pressure (MAPopt) has been increasingly evaluated in contexts where intracranial pressure (ICP) monitoring is not feasible, such as cardiac surgery (Tabone et al., 2024), cardiac arrest (Sekhon et al., 2016), aneurysmal subarachnoid hemorrhage (aSAH) (Carlson et al., 2025), and neonatal hypoxic–ischemic encephalopathy (HIE) (Howlett et al., 2013). MAPopt is commonly derived from transcranial Doppler (TCD)‐ or near‐infrared spectroscopy (NIRS)‐based indices and may serve as a noninvasive alternative to invasive methods in clinical settings. Finally, a recent concept is the optimal bispectral index (BISopt), which represents the level of sedation associated with the most stable cerebral autoregulation. BISopt has been proposed as a potential complement to perfusion‐based targets, particularly in perioperative and TBI populations, though its role remains poorly defined (Froese, Gomez, Sainbhi, Batson, Stein, et al., 2022; Stein et al., 2025).

These optimal (Opt) parameters are derived from continuous monitoring of cerebrovascular reactivity indices, such as the PRx, cerebral oximetry index (COx), in combination with either blood pressure or electroencephalography data sources. By applying curve‐fitting techniques, most commonly a second‐order polynomial fit to the relationship between physiologic parameters (e.g., PRx vs. CPP), Opt parameters identify the nadir of the U‐shaped autoregulatory curve (Beqiri et al., 2023). This nadir corresponds to the pressure or sedation depth at which autoregulatory function is most intact, providing a patient‐specific “optimal” target (Aries et al., 2012; Steiner et al., 2002).

Although these optimal parameters share a common conceptual framework, the autoregulation proxy indices from which they are derived differ considerably (Highton et al., 2015). PRx, PAx, COx, THx, ORx, and related indices vary in their underlying physiologic signals (pressure, flow velocity, and oxygenation), analytic bandwidths, and susceptibility to artifacts (Calviello et al., 2020; Rivera‐Lara et al., 2020). Consequently, they may yield divergent Opt estimates even within the same patient or time period. Differences in the duration of availability, coherence, and power spectra further contribute to variability across studies (Bögli, Olakorede, Beqiri, Cucciolini, et al., 2025; Eide et al., 2007). From a physiologic standpoint, even within the same personalized target (e.g., CPPopt), multiple Opt values may coexist depending on the autoregulation index used for their derivation. For instance, PRx‐, PAx‐, or COx‐based CPPopt estimates obtained at a given time point for the same patient may differ, each reflecting distinct facets of cerebrovascular reactivity and offering complementary insights into autoregulatory behavior (Dias et al., 2015; Stein et al., 2023).

Despite growing interest, a major challenge lies in the heterogeneity of methodologies and monitoring modalities used to derive these Opt parameters. CPPopt can be calculated from ICP‐derived indices such as PRx, PAx, and RAC (Stein et al., 2023; Zeiler et al., 2019), but also from NIRS‐ and brain tissue oxygenation‐based measures such as COx, THx, and ORx (Dias et al., 2015). MAPopt derivation is equally variable, incorporating TCD‐based Mx (Blaine Easley et al., 2013; Liu et al., 2022; Rivera‐Lara et al., 2017), NIRS‐based HVx/TOx/COx/COx_a (Bindra et al., 2016; Blaine Easley et al., 2013; Hoiland et al., 2020; Liu et al., 2021; Oshorov et al., 2022; Silverman et al., 2019), and more recently skeletal muscle oxygenation indices (Mirsajadi et al., 2024). Similarly, BISopt has been explored using both PRx‐ and NIRS‐based approaches (Froese, Gomez, Sainbhi, Batson, Slack, et al., 2022; Froese, Gomez, Sainbhi, Batson, Stein, et al., 2022). This diversity raises critical questions about the interchangeability, reliability, and clinical relevance of Opt parameters across indices. The objective of this scoping review is therefore to systematically synthesize the evidence comparing CPPopt, MAPopt, and BISopt across different autoregulatory indices. By examining convergences, divergences, and methodological considerations, this review aims to clarify the current state of knowledge and identify priorities for future research.

## 2 METHODS

This systematic scoping literature review was conducted following the Cochrane Handbook for Systematic Reviews methodology (Page et al., 2021). This review followed the Preferred Reporting Items for Systematic Reviews and Meta‐Analyses extension for Scoping Reviews (PRISMA‐ScR) (Page et al., 2021; Tricco et al., 2018). The completed PRISMA‐ScR checklist can be found in Table S1. The objectives for this systematic scoping review search strategy were developed collaboratively by RH and FAZ.

### 2.1 Ethical consideration

All articles included in this systematic scoping review were previously published in peer‐reviewed journals and had already undergone ethical screening by those outlets. Accordingly, no additional ethics approval was required for this review.

### 2.2 Search question/objective and criteria for inclusion and exclusion

Search objective of this review is to identify all existing literature describing the relationship between CPPopt/MAPopt/BISopt derived from different autoregulatory indices. To be included in this review, studies were required to report relationships between CPPopt, MAPopt, or BISopt, whether comparing the same parameter across different autoregulatory indices (e.g., CPPopt‐CPPopt, MAPopt‐MAPopt, and BISopt‐BISopt) or cross‐comparisons between parameters derived from different/same autoregulatory indices (e.g., CPPopt‐MAPopt, BISopt‐CPPopt, and BISopt‐MAPopt). If Opts were compared based on derivation method or any other criteria other than autoregulatory indices, then those relationships or studies were not considered. Additionally, non‐peer‐reviewed or non‐full‐length articles (e.g., abstracts, theses, conference papers, and book chapters), non‐English articles and non‐human studies were excluded.

### 2.3 Search strategy

A literature search was conducted to identify studies evaluating the relationship between different Opt parameters. The search strategy was applied across BIOSIS, SCOPUS, EMBASE, MEDLINE, Global Health, and the Cochrane Library, from database inception to May 12, 2025. Search terms included MAPopt, ABPopt, BISopt, and relevant synonyms; the complete search string is provided in Appendix. Following database searches, results were consolidated and deduplicated to generate a comprehensive list of potential articles.

### 2.4 Selecting studies

Following deduplication, articles underwent a two‐stage, dual‐reviewer screening process. In the first stage, two reviewers (RH and KZ) independently assessed titles and abstracts against the predefined inclusion and exclusion criteria. Articles deemed eligible proceeded to the second stage, where both reviewers evaluated the full texts using the same criteria. Discrepancies were resolved through consultation with a third reviewer (FAZ). Finally, the reference lists of all included studies were screened to identify any additional relevant publications not captured in the initial search.

### 2.5 Data collection

Characteristics were recorded from each article included in this systematic scoping review, encompassing patient/subject information, general study information, and results. Patient/subject information included the patient cohort investigated, patient demographics, and underlying pathology used in the study. General study information included the primary study objective, technology used for CVR assessment, and CVR index used for Opt derivation. Results included the relationship between different Opt parameters.

### 2.6 Statistical analysis

Considering the highly heterogeneous nature of the literature included, no formal meta‐analysis was performed.

### 2.7 Bias assessment

As the primary objective of this review was to provide a comprehensive scoping synthesis of the available literature, which had already undergone peer‐review screening by their respective journals, a formal risk of bias assessment was not deemed necessary.

## 3 RESULTS

### 3.1 Search strategy and results

**FIGURE 1:**
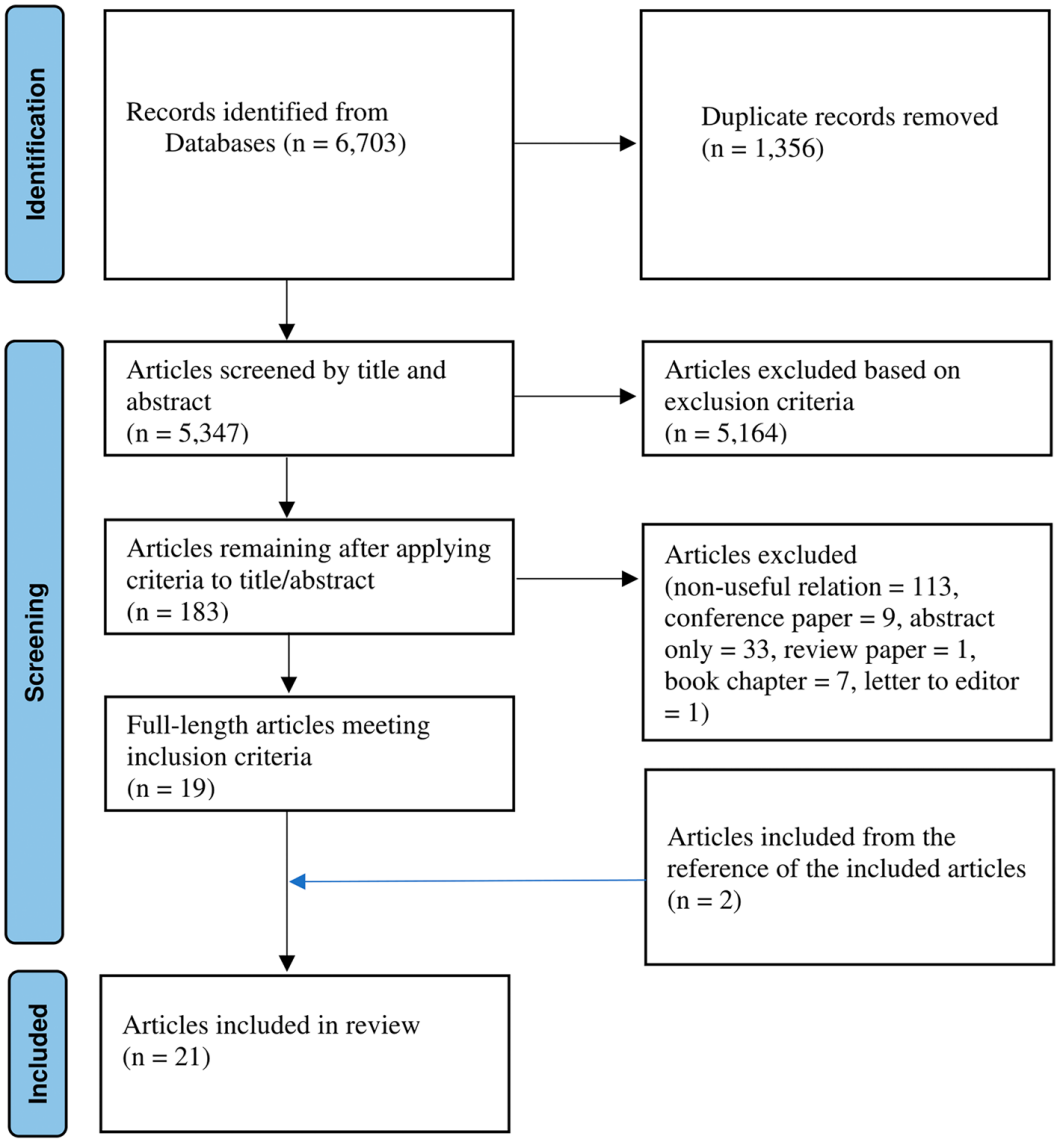
PRISMA flow diagrams of the systematically conducted scoping review.

The initial search yielded 6703 articles, of which 5347 remained after deduplication. These unique records were screened by title and abstract, resulting in the exclusion of 5164 articles that did not meet the eligibility criteria. The remaining 183 articles underwent full‐text review, from which 164 were excluded, leaving 19 articles for inclusion. An additional two articles were identified through reference list screening, bringing the total number of included studies to 21. The Figure 1 presents the overall search and screening results using a PRISMA flow diagram.

### 3.2 Study demographics

Among the 21 studies, 10 of them included the relationship between CPPopt derived from different autoregulatory indices (Depreitere et al., 2014; Dias et al., 2015; Lang et al., 2015; Liu et al., 2017; Radolovich et al., 2009; Riemann et al., 2020; Santos et al., 2011; Stein et al., 2023; Zeiler et al., 2019; Zweifel et al., 2010), nine of them included the relationship between MAPopt derived from different autoregulatory indices (Bindra et al., 2016; Blaine Easley et al., 2013; Hoiland et al., 2020; Hori et al., 2015; Liu et al., 2022; Oshorov et al., 2022; Rivera‐Lara et al., 2017; Silverman et al., 2019; Zweifel et al., 2010), one study included the relationship between BISopt derived from different autoregulatory indices (Froese, Gomez, Sainbhi, Batson, Slack, et al., 2022), one study included the relationship between BISopt and CPPopt (Froese, Gomez, Sainbhi, Batson, Stein, et al., 2022), and one study included the relationship between BISopt and MAPopt (Froese et al., 2024). One study included both CPPopt versus CPPopt, and MAPopt versus MAPopt relationship (Zweifel et al., 2010). Table 1 lists studies that show the relationship between CPPopt and CPPopt, Table 2 lists studies that show the relationship between MAPopt and MAPopt, Table 3 lists studies that show the relationship between BISopt and BISopt, and Table 4 lists studies that show the relationship between cross‐Opts.

**TABLE 1:** Relationships between CPPopt derived from different autoregulatory indices.

Article	Patient cohort characteristics and study site	Primary objective of study	Underlying pathology	Technology used for CVR assessment	CVR index used for opt derivation	Relationship between Opts derived from different indices	
Depreitere et al. (2014)	264 TBI patient (22 neuro‐ICUs in 11 European countries between March 2003 and July 2005) were included.	Examined how processing routine minute‐by‐minute ICP and MABP data can be improved to generate CPPopt recommendations that align with those derived from the PRx method, demonstrate similar associations with patient outcomes, and can be produced during over two‐thirds of the monitoring period.	TBI	ICP monitoring	PRx and LAx LAx values were calculated for varying time windows. Did not mention whether the relationship assessed between indices was based on overall average or point‐by‐point average. Automatic curve‐fitting was used for opt calculation.	Correlation: No statistically significant difference was observed between the per‐patient median CPPopt values derived from DATACAR and PRx (*p* = 0.802), with medians of 72.5 mmHg (IQR 62.5–77.5 mmHg) and 72.5 mmHg (IQR 67.5–77.5 mmHg), respectively. The DATACAR method generated a CPPopt recommendation during a median of 97% (IQR 94%–98%) of the monitoring period, whereas the PRx‐based method provided recommendations for a median of only 44% (IQR 31%–55%).	Bias:
Dias et al. (2015)	18 multiple‐trauma patients with severe TBI admitted to the NCCU at Hospital Sao Joao, Porto, Portugal	Assessed the impact on clinical outcomes of a novel autoregulation‐guided treatment (CPPopt) derived from continuous monitoring of cerebrovascular reactivity (PRx).	TBI	ICP monitoring, NIRS	PRx, COx, ORx: Oxygen Reactivity Index, from brain tissue oxygenation, CBFx: Cerebral Blood Flow Index, from thermal diffusion CBF sensor Autoregulatory indices were calculated using a 5‐min‐long moving window, updated every minute. Did not mention whether the relationship assessed between indices was based on overall average or point‐by‐point average. Automatic curve‐fitting was used for Opt calculation.	Correlation: Among the indices compared, COx‐CPPopt performed best in terms of agreement with the reference PRx‐derived CPPopt. All other indices had lower yield for CPPopt than PRx (29% for ORx_CPPopt, 22% for COx_CPPopt and 23% for CBFx_CPPopt versus 59% for PRx_CPPopt).	Bias: Among the indices compared, COx‐CPPopt had the smallest average bias (−0.1 mmHg) in respect to PRx‐CPPopt. However, all alternative indices (ORx and CBFx) had slightly higher average bias (+3.7 mmHg, 4.5 mmHg respectively). Despite this, their similar limit of precision (6.3 mmHg, 6.7 mmHg and 6.0 mmHg for COx, ORx, and CBFx, respectively) suggests potential utility, especially in settings where invasive monitoring is limited.
Lang et al. (2015)	307 patients (77% male) with TBI were hospitalized and treated. The Neurocritical Care Unit, Addenbrooke's Hospital, University of Cambridge, United Kingdom	Evaluated the utility and performance of the long pressure reactivity index (L‐PRx), compared with the conventional short pressure reactivity index (PRx), for long‐term outcome assessment and optimal cerebral perfusion pressure (CPPopt) calculation in a large cohort of patients with TBI.	TBI	ICP monitoring	PRx, L‐PRx Autoregulatory indices were calculated using 5‐min‐long window for PRx, and 20‐ min‐long window for L‐PRx. The relationship assessed between indices was based on overall average. Automatic curve‐fitting method was used for Opt calculation.	Correlation: No significant difference was observed between CPPopt values derived from PRx and L‐PRx (median 74.7 mmHg, IQR ± 8.2 mmHg vs. 76.9 mmHg, IQR ± 10.1 mmHg). For pooled data, CPPopt was approximately 5 mmHg higher when calculated with L‐PRx compared to PRx. Mortality was associated with mean CPP values below the PRx‐derived CPPopt (χ^2^ = 30.6, *p* < 0.00001), while severe disability was associated with mean CPP values above the PRx‐derived CPPopt (χ^2^ = 7.8, *p* = 0.005). These associations were not statistically significant when CPPopt was calculated using L‐PRx.	Bias:
Liu et al. (2017)	515 patients (385 male) with TBI were recruited. Addenbrooke's Hospital, University of Cambridge, United Kingdom	Aimed to introduce and assess wPRx for TBI patients, comparing it to the traditional PRx in terms of stability, CPPopt estimation, and outcome prediction.	TBI	ICP monitoring	PRx, wPRx PRx was calculated using a 5‐min‐long moving window. The relationship assessed between indices was based on hour‐by‐hour average data. Automatic curve‐fitting method was used for Opt calculation.	Correlation: The study found a strong positive correlation between CPPopt derived from PRx and that from wPRx (*r* = 0.81, *p* < 0.001), indicating that the two methods are related and track similar underlying physiological phenomena. CPPopt_wPRx was more stable (lower standard deviation: 7.05 ± 3.78 vs. 8.45 ± 2.90 for CPPopt_PRx, *p* < 0.001). CPPopt_wPRx had a higher yield, meaning it could be calculated for a greater proportion of the monitoring time (59.6% ± 27% vs. 53.2% ± 20%, *p* < 0.001).	Bias: The Bland–Altman analysis showed high agreement between the two CPPopt methods, with a small overall bias. This implies that both methods provide comparable estimates of CPPopt, although not identical.
Radolovich et al. (2009)	32 head injury patients were included. Neurosciences Critical Care Unit of Addenbrooke's Hospital, Cambridge, UK	Aimed to compare similar indices describing interaction between changes in intracranial pressure (ICP), arterial blood pressure (ABP), and brain tissue oxygen to verify their clinical utility in patients after traumatic brain injury	Head injury	ICP monitoring	PRx, ORx PRx was calculated using a 5‐min‐long moving window, updated every minute. ORx was calculated over an average period of 1 h, updated every minute. Did not mention whether the relationship assessed between indices was based on overall average or point‐by‐point average.	Correlation: The PRx_CPPopt and the ORx_CPPopt did not correlate with each other (*r* = 0.07; *p* > 0.056; *N* = 14). ‐Did not mention the method used for Opt calculation.	Bias:
Riemann et al. (2020b)	224 TBI (176 males) patients were included in this study. Took place at 21 centers across the European Union	Examined the discriminative value of a low‐resolution long pressure reactivity index (L‐PRx) and its derived “optimal CPP” in comparison with the established high‐resolution PRx.	TBI	ICP monitoring	PRx, L‐PRx PRx was calculated using a 5‐min‐long moving window, updated every minute. L‐PRx was calculated using a 20‐min‐long moving window, updated every minute. The relationship assessed between indices was based on minute‐by‐minute average data. Automatic curve‐fitting method was used for Opt calculation.	Correlation: CPPopt values derived from L‐PRx and PRx were comparable (median 71.4 mmHg [65.9–76.6] vs. 72.0 mmHg [65.9–77.5], *p* = 0.445). Similarly, CPPopt yield did not differ significantly between the two methods (80.0% [70.4–86.7] vs. 80.4% [71.4–87.6], *p* = 0.625).	Bias:
Santos et al. (2011)	18 patients suffering from nontraumatic intracerebral hemorrhage (ICH) were included in the study.	Compared conventional PRx with low‐frequency PRx (L‐PRx), a method designed to exclude rapid fluctuations of MAP and ICP by filtering out signals with frequencies greater than 0.01 Hz.	Nontraumatic intracerebral hemorrhage (ICH)	ICP monitoring	PRx, L‐PRx PRx was calculated using a 5‐min‐long moving window. L‐PRx was calculated using a 20‐min‐long moving window. The relationship assessed between indices was based on averaged entire recording period data.	Correlation: The correlation between PRx_CPPopt and L‐PRx_CPPopt was high (*r* = 0.980, *p* < 0.001, *N* = 7). Inspection and binning method was used for Opt calculation.	Bias:
Zeiler et al. (2019)	93 adult patients (73 males) with TBI were included. Winnipeg Health Sciences Centre, Canada.	Evaluated CPPopt parameters derived from three ICP‐based cerebrovascular reactivity indices and determined which parameter most accurately predicts 6‐ to 12‐month clinical outcomes.	TBI	ICP monitoring	PRx, PAx (Pulse amplitude Index), RAC (R = correlation (standard statistics symbol), A = AMP, C = CPP) Autoregulatory indices were calculated using a 5‐min‐long moving window, updated every minute. Automatic curve‐fitting method was used for Opt calculation.	Correlation: Mean PRx‐based CPPopt 71.7 mm Hg (SD 9.3), Mean PAx‐based CPPopt 69.0 mm Hg (SD 11.2), Mean RAC‐based CPPopt 68.9 mm Hg (SD 9.9). Mean CPPopt values are very similar across CVR indices.	Bias:
Stein et al. (2023)	103 patients (81% male) with moderate to severe TBI were included. Winnipeg Acute TBI Database	Investigated the association between various metrics of CPPopt and failure to improve in outcome over time.	TBI	ICP monitoring	PRx, PAx, RAC Autoregulatory indices were calculated using a 5‐min‐long moving window, updated every minute. Automatic curve‐fitting method was used for Opt calculation.	Correlation: Median PRx‐based CPPopt 74.30 mm Hg (IQR 69.29–80.02) Median PAx‐based CPPopt 74.97 mm Hg (IQR 71.64–82.50), Median RAC‐based CPPopt 73.24 mm Hg (IQR 69.48–79.30). Median CPPopt values are very similar across CVR indices.	Bias:
Zweifel et al. (2010)	40 patients with close‐head injury were included. Neurosciences Critical Care Unit at Addenbrooke's Hospital (Cambridge University Hospitals NHS Foundation Trust)	Compared a NIRS‐based cerebrovascular reactivity index, total hemoglobin reactivity (THx), with standard PRx measurements.	Close‐head injury	ICP monitoring and NIRS	PRx by ICP monitoring, THx by NIRS Autoregulatory indices were calculated using a 5‐minute‐long moving window. The relationship assessed between indices was based on averaged entire recording period data per patient. Inspection and binning method was used for Opt calculation.	Correlation: A significant correlation was observed between CPPopt values assessed by PRx and THx across 50 recordings (r = 0.74, *p* < 0.0001), with no significant difference detected in a paired comparison using the signed‐rank test (*p* = 1.0).	Bias: The mean bias between PRx‐ and THx‐derived CPPopt was 4.5 mmHg.

Abbreviations: CBF, cerebral blood flow; CBFx, cerebral blood flow index; COx, cerebral oximetry index; CPPopt, optimal cerebral perfusion pressure; DATACAR, dynamic adaptive target of active cerebral autoregulation; ICP, intracranial pressure; IQR, interquartile range; LAx, low‐frequency autoregulation index; L‐PRx, low resolution long pressure reactivity index; NIRS, near infrared spectroscopy; ORx, oxygen reactivity index; PAx, pulse amplitude index; PRx, pressure reactivity index; RAC, (R, = correlation (standard statistics symbol), A = AMP, C = CPP); SD, standard deviation; TBI, traumatic brain injury; THx, total hemoglobin reactivity index; wPRx, wavelet‐based pressure reactivity index.

**TABLE 2:** Relationships between MAPopt/ABPopt derived from different autoregulatory indices.

Article	Patient cohort characteristics and study site	Primary objective of study	Underlying pathology	Technology used for CVR assessment	CVR index used for opt derivation	Relationship between opts derived from different indices	
Blaine Easley et al. (2013)	109 adult patients (73.4% males) for cardiopulmonary bypass were included in the study. John Hopkins university	Hypothesized that the lower limit of autoregulation (LLA) and the optimal arterial blood pressure (ABPopt), reflecting the point of most effective autoregulation, could be identified using HVx in patients undergoing CPB	Cardiopulmonary bypass (CPB)	NIRS, TCD	Mx from TCD, HVx from NIRS Autoregulatory indices were calculated using a 5‐min‐long moving window, updated every 10 s. The relationship assessed between indices was based on averaged entire recording period data per patient. Inspection and binning method was used for opt calculation.	Correlation: The mean ABPopt was 75 ± 11 mmHg (IQR 65–80 mmHg) using mx and 74 ± 13 mmHg (IQR 65–85 mmHg) using HVx. A significant correlation and agreement were observed between the two methods (Pearson *r* = 0.592, *p* < 0.0001).	Bias: Bland–Altman bias = 1.31 mmHg, 95% limits of agreement −19 to 22 mmHg.
Hoiland et al. (2020)	10 patients (7 male) with cardiac arrest were included. Vancouver General Hospital	Determined the agreement between PRx‐derived MAPopt versus NIRS‐derived MAPopt.	Hypoxic ischemic brain injury (HIBI)	ICP monitoring, NIRS	PRx, COx_a Did not mention the window size or update frequency for autoregulatory index calculation. Did not mention whether the relationship assessed between indices was based on overall average or point‐by‐point average. Did not clearly specify the exact Opt parameter derivation method.	Correlation: MAPopt values derived from PRx and COx_a demonstrated a significant correlation (*R* = 0.39, *p* < 0.01).	Bias: The mean bias between PRx‐ and COx_a‐derived MAPopt was 1.4 mmHg, indicating a slight underestimation when using COx_a. However, the limits of agreement were relatively wide, ranging from −23.0 mmHg to 25.9 mmHg.
Hori et al. (2015)	64 patients (38 male) undergoing Cardiopulmonary bypass (CPB) were included. John Hopkins University	Evaluated the accuracy of cerebral autoregulation monitoring using microcirculatory flow assessed with innovative ultrasound‐tagged near‐infrared spectroscopy (UT‐NIRS) compared with transcranial Doppler (TCD).	CPB	Ultrasound tagged NIRS (UT‐NIRS), TCD	Mx from TCD, CFVx from UT‐NIRS Autoregulatory indices were calculated using a 5‐min‐long moving window. The relationship assessed between indices was based on averaged entire recording period data per patient. Did not clearly specify the exact Opt parameter derivation method.	Correlation: The mean MAPopt was 74 ± 12 mmHg based on Mx and 71 ± 12 mmHg based on CFVx (*p* = 0.0514). During CPB, MAPopt measured by Mx and CFVx showed a strong correlation (*r* = 0.71, 95% CI 0.56–0.81, *p* < 0.0001).	Bias: Mean bias of −2.85 ± 8.54 mmHg and 95% limits of agreement ranging from −19.60 to 13.89 mmHg.
Oshorov et al. (2022)	3 male patients with severe TBI were included. Burdenko Neurosurgery Institute, Russia	Compared regional oxygen saturation (rSO_2_)‐based CA (COx_a) with ICP/ABP‐based CA (PRx) in TBI patients and compared MAPopt derived from both technologies.	Severe TBI	NIRS, ICP monitoring	PRx, COx_a Autoregulatory indices were calculated using a 5‐min‐long moving window. The relationship assessed between indices was based on averaged entire recording period data per patient. Did not clearly specify the exact Opt parameter derivation method.	Correlation: Correlation between MAPopt calculated for COx and PRx was *r* = 0.49, *p* < 0.038.	Bias: Good agreement with a bias of 0.39 ± 7.89 for COx_MAPopt versus PRx_MAPopt.
Rivera‐Lara et al. (2017)	33 comatose patients (21 females) were included. The neurocritical care unit (NCCU) at the Johns Hopkins Hospital	Assessed the accuracy of rSO_2_‐based cerebral autoregulation monitoring compared with transcranial Doppler (TCD) in comatose patients with acute neurologic injury.	under coma	NIRS, TCD	COx_a by NIRS, Mx by TCD Autoregulatory indices were calculated using a 5‐min‐long moving window. The relationship assessed between indices was based on the entire recording period data per patient. Did not clearly specify the exact Opt parameter derivation method.	Correlation: The median MAPopt was 85 mmHg (IQR 80–107.5 mmHg) when calculated using COx and 90 mmHg (IQR 80–100 mmHg) when calculated using Mx. These measures demonstrated a strong correlation (Pearson r = 0.87, *p* < 0.001).	Bias:
Silverman et al. (2019)	31 patients (23 females) with aneurysmal subarachnoid hemorrhage (aSAH) were included. Yale‐New Haven Hospital Emergency Department, Connecticut, USA	Calculated personalized blood pressure targets at which cerebral autoregulation was best preserved and analyzed how deviation from these limits correlates with functional outcome.	aSAH	NIRS, ICP monitoring	TOx by NIRS, PRx by ICP monitoring Did not mention the window size or update frequency for autoregulatory index calculation. Did not mention whether the relationship assessed between indices was based on overall average or point‐by‐point average. Automatic curve‐fitting was used for Opt calculation.	Correlation: NIRS‐ and ICP‐derived MAPopt demonstrated strong correlations with one another (Spearman coefficient = 0.93, P < 0.0001).	Bias: Bland–Altman analyses of NIRS‐ vs. ICP‐derived MAPopt found a bias of −0.7 (standard deviation = 5.1), which demonstrates agreement between these modalities.
Bindra et al. (2016)	19 patients (7 females) with different acute pathologies (sepsis, cardiac arrest, head injury, stroke) were included. Intensive care unit (ICU) of Liverpool Hospital, New South Wales, Australia	Evaluated the agreement between a NIRS‐based autoregulation index derived from invasive blood pressure monitoring and a fully non‐invasive autoregulation index obtained from continuous non‐invasive blood pressure (nABP) monitoring using the Finometer photoplethysmograph.	Different acute pathologies (sepsis, cardiac arrest, head injury and stroke)	NIRS, ICP monitoring	iTOx, nTOx Autoregulatory indices were calculated using a 5‐min‐long moving window. The relationship assessed between indices was based on the averaged entire recording period data. Automatic curve‐fitting method was used for Opt calculation.	Correlation: Simultaneous calculation of iABPopt and nABPopt was possible in 38 of 102 recordings (37%). In these recordings, iABPopt and nABPopt demonstrated a moderate correlation (*r* = 0.47, *p* = 0.003).	Bias: Mean bias of 5.8 mmHg and 95% limits of agreement ±32.1 mmHg.
Liu et al. (2022)	240 patients (191 males) with cardiac surgery were included. Johns Hopkins University.	Compared the association of several cerebral autoregulation metrics, calculated using different methods, with outcomes after cardiac surgery.	Cardiac Surgery	NIRS, TCD	Mx by TCD, COx_a by NIRS Autoregulatory indices were calculated using a 5‐min‐long moving window. The relationship assessed between indices was based on the averaged entire recording period data per patient. Automatic curve‐fitting method was used for Opt calculation.	Correlation: Mean COx_MAPopt = 76.8 mm Hg (SD 8.7) Mean Mx_MAPopt = 75.5 mm Hg (SD 8.6) Mean MAPopt values are very similar across CVR indices.	Bias:
Zweifel et al. (2010)	40 patients with close‐head injury were included. Neurosciences Critical Care Unit at Addenbrooke's Hospital (Cambridge University Hospitals NHS Foundation Trust)	Compared a NIRS‐based cerebrovascular reactivity index, total hemoglobin reactivity (THx), with standard PRx measurements.	Close‐head injury	ICP monitoring and NIRS	PRx, THx by NIRS Autoregulatory indices were calculated using a 5‐min‐long moving window. The relationship assessed between indices was based on averaged entire recording period data per patient. Inspection and binning method was used for Opt calculation.	Correlation: ABPopt values assessed by PRx and THx were significantly correlated across 53 recordings (*r* = 0.82, *p* < 0.0001), with no significant difference observed in a paired signed‐rank test (*p* = 1.0).	Bias: The mean bias between PRx‐ and THx‐derived ABPopt was 4.06 mmHg.

Abbreviations: ABPopt, optimal arterial blood pressure; aSAH, aneurysmal subarachnoid hemorrhage; CA, cerebral autoregulation; CFVx, cerebral flow velocity index; COx, cerebral oximetry index; COx_a, cerebral oximetry index based on arterial blood pressure; CPB, cardiopulmonary bypass; CPPopt, optimal cerebral perfusion pressure; HIBI, hypoxic ischemic brain injury; HVx, hemoglobin volume index; iABPopt, invasive optimal arterial blood pressure; ICP, intracranial pressure; IQR, interquartile range; iTOx, invasive autoregulation index; MAPopt, optimal mean arterial blood pressure; Mx, mean velocity index; nABPopt, entirely noninvasive optimal arterial blood pressure; NIRS, near infrared spectroscopy; nTOx, entirely noninvasive autoregulation index; PRx, pressure reactivity index; SD, standard deviation; TBI, traumatic brain injury; TCD, transcranial doppler; THx, total hemoglobin reactivity index; TOx, tissue oxygenation index.

**TABLE 3:** Relationships between BISopt derived from different autoregulatory indices.

Article	Patient cohort characteristics and study site	Primary objective of study	Underlying pathology	Technology used for CVR assessment	CVR index used for opt derivation	Relationship between opts derived from different indices	
Froese, Gomez, Sainbhi, Batson, Slack, et al. (2022)	42 patients (86 percent male) with moderate to severe TBI were included. Health Sciences Centre, Winnipeg, University of Manitoba, Winnipeg, Manitoba, Canada	Assessed the relationship between objectively measured depth of sedation through BIS and autoregulatory capacity measured through COx_a.	Moderate‐to‐severe TBI	NIRS, ICP monitoring	COx_a by NIRS, PRx PRx was calculated using a 5‐min‐long moving window, updated every minute. COx_a was calculated using a 5‐min‐long moving window, updated every minute. The relationship assessed between indices was based on the entire recording period data per patient. Automatic curve‐fitting method was used for opt calculation.	Correlation: The median COx_a‐derived BISopt was 48 (IQR: 40–56), compared with a median PRx‐derived BISopt of 45 (IQR: 40–56). This difference was not statistically significant (Wilcoxon signed‐rank test, *p* = 0.31).	Bias:

Abbreviations: BISopt, optimal bispectral index; COx_a, cerebral oximetry index based on arterial blood pressure; IQR, interquartile range; NIRS, near infrared spectroscopy; PRx, pressure reactivity index; TBI, traumatic brain injury.

**TABLE 4:** Showing relationships between BISopt and other Opts.

Article	Patient cohort characteristics and study site	Primary objective of study	Underlying pathology	Technology used for CVR assessment	CVR index used for opt derivation	Relationship between opts derived from different indices	
Froese, Gomez, Sainbhi, Batson, Stein, et al. (2022)	32 patients (87.5% male) with moderate to severe TBI were included. Health Sciences Centre, Winnipeg, University of Manitoba, Winnipeg, Manitoba, Canada	Developed a methodology to derive optimal bispectral index (BISopt) values in patients with moderate to severe TBI by integrating continuous monitoring of CVR with bispectral electroencephalography.	Moderate‐to‐severe TBI	ICP monitoring	PRx PRx was calculated using a 5‐min‐long moving window, updated very minute. Automatic curve‐fitting method was used for Opt calculation.	Correlation: No correlation was found between BISopt and CPPopt (*r* = 0.15, *p* = 0.38) derived over the entire recording period.	Bias:
Froese et al. (2024)	57 Healthy controls and 27 elective surgery patients were included in this study. Health Sciences Centre, Winnipeg, University of Manitoba, Winnipeg, Manitoba, Canada	Investigated the relationship between BIS and continuous cerebrovascular reactivity in two cohorts: (A) patients undergoing elective spinal surgery under general anesthesia, and (B) awake healthy volunteers.	No pathology	NIRS	COx_a COx_a was calculated using a 5‐min‐long moving window, updated every minute. Both automatic curve‐fitting and visual inspection method were used for Opt calculation.	Correlation: Limited identifiable relationship found between BISopt and MAPopt	Bias:

Abbreviations: BISopt, optimal bispectral index; COx_a, cerebral oximetry index based on arterial blood pressure; CPPopt, optimal cerebral perfusion pressure; CVR, cerebrovascular reactivity; ICP, intracranial pressure; IQR, interquartile range; MAPopt, optimal mean arterial pressure; NIRS, near infrared spectroscopy; PRx, pressure reactivity index; TBI, traumatic brain injury.

#### 3.2.1 CPPopt versus CPPopt (Table 1)

Ten studies evaluated the relationship between CPPopt derived from different autoregulatory indices, predominantly in traumatic brain injury (TBI) populations. Except for Dias et al. (2015), all other TBI studies had close to 100 or more than 100 subjects per study. Across these studies, median CPPopt values consistently clustered within the 70–76 mmHg range, but agreement varied depending on the index and analytic approach.

There were several studies that demonstrated strong agreement between PRx‐derived CPPopt and alternative ICP‐based indices. Zeiler et al. (2019) and Stein et al. (2023) found near‐identical CPPopt values when calculated from PRx, PAx, and RAC, with differences <3 mmHg. Lang et al. (2015) and Riemann et al. (2020) similarly reported no significant differences between CPPopt derived from PRx and long, low frequency PRx (L‐PRx). Regarding calculating L‐PRx, these two studies used slightly different methods, but that did not impact the outcome. In another study, Santos et al. (2011) reported a stronger correlation between PRx_CPPopt and L‐PRx_CPPopt compared to Riemann et al. (2020) (*r* = 0.98 vs. *r* = 0.445) (Riemann et al., 2020; Santos et al., 2011). This discrepancy may partly be explained by the considerably larger sample size in Riemann et al. (2020) study relative to that of Santos et al. (2011). The study by Depreitere et al. (2014) introduced a novel CVR index called low‐frequency autoregulation index (LAx) and demonstrated that when applied with the multi‐window DATACAR (Dynamic Adaptive Target of Active Cerebral Autoregulation) method, it produced CPPopt values comparable to those derived from PRx (median 72.5 mmHg) but delivered recommendations with much greater consistency (97% vs. 44%) of monitoring time.

Signal processing refinements improved CPPopt derivation in some cases. Liu et al. (2017) reported strong correlation between PRx‐ and wPRx‐derived CPPopt (*r* = 0.81, *p* < 0.001), with wPRx (wavelet based) showing improved yield (59.6% vs. 53.2%) and stability.

Comparisons with noninvasive or alternative physiologic indices were mixed. Zweifel et al. (2010) showed a significant correlation between PRx‐ and NIRS‐derived THx CPPopt (*r* = 0.74, bias 4.5 mmHg). Dias et al. (2015) demonstrated that NIRS‐based COx‐derived CPPopt most closely approximated invasive PRx‐derived CPPopt. However, indices based on brain tissue oxygenation (ORx) and cerebral blood flow (CBFx) yielded weaker agreement with PRx, but all three indices (COx, ORx, and CBFx) had a similar limit of precision which indicates their potential utility when invasive monitoring is unavailable. Importantly, Radolovich et al. (2009) found no correlation between PRx‐ and ORx‐derived CPPopt (*r* = 0.07), highlighting that some indices may not reflect equivalent physiologic processes.

A summary table showing the results regarding the correlation between the same Opt parameter derived from different autoregulatory indices is given below (Table 5).

**TABLE 5:** Showing whether there is any significant relation present between the same Opt parameter derived from different autoregulatory indices.

Autoregulatory indices and specific opt parameter	Correlation assessed?	Significant positive correlation found?	Studies
PRx and LAx: CPPopt	Yes	Yes	Depreitere et al. (2014)
PRx and COx: CPPopt	Yes	Yes	Dias et al. (2015)
PRx and L‐PRx: CPPopt	Yes	Yes	Lang et al. (2015), Riemann et al. (2020), and Santos et al. (2011)
PRx and wPRx: CPPopt	Yes	Yes	Liu et al. (2017)
PRx and ORx: CPPopt	Yes	No	Radolovich et al. (2009)
PRx and PAx: CPPopt	No	No	Zeiler et al., 2019 and Stein et al. (2023)
PRx and RAC: CPPopt	No	No	Zeiler et al. (2019) and Stein et al. (2023)
PRx and THx: CPPopt	Yes	Yes	Zweifel et al. (2010)
HVx and Mx: MAPopt/ABPopt	Yes	Yes	Blaine Easley et al. (2013)
PRx and COX_a: MAPopt/ABPopt	Yes	Yes	Hoiland et al. (2020); Oshorov et al. (2022)
Mx and CFVx: MAPopt/ABPopt	Yes	Yes	Hori et al. (2015)
Mx and COx_a: MAPopt/ABPopt	Yes	Yes	Rivera‐Lara et al. (2017)
PRx and TOx: MAPopt/ABPopt	Yes	Yes	Silverman et al. (2019)
iTOx and nTOx: MAPopt/ABPopt	Yes	Yes	Bindra et al. (2016)
PRx and THx: MAPopt/ABPopt	Yes	Yes	Zweifel et al. (2010)
PRx and COx_a: BISopt	Yes	Yes	Froese, Gomez, Sainbhi, Batson, Slack, et al. (2022)

Abbreviations: ABPopt, optimal arterial blood pressure; BISopt, optimal bispectral index; CBFx, cerebral blood flow index; CFVx, cerebral flow velocity index; COx, cerebral oximetry index; COx, cerebral oximetry index; COx_a, cerebral oximetry index based on arterial blood pressure; CPPopt, optimal cerebral perfusion pressure; DATACAR, dynamic adaptive target of active cerebral autoregulation; HVx, hemoglobin volume index; iTOx, invasive autoregulation index; LAx, low‐frequency autoregulation index; L‐PRx, low resolution long pressure reactivity index; MAPopt, optimal mean arterial blood pressure; MOx, muscle tissue saturation index; MVx, muscle total hemoglobin index; Mx, mean velocity index; nTOx, entirely noninvasive autoregulation index; ORx, oxygen reactivity index; PAx, pulse amplitude index; PRx, pressure reactivity index; RAC, (R = correlation (standard statistics symbol), A = AMP, C = CPP); THx, total hemoglobin reactivity index; THx, total hemoglobin reactivity index; TOx, tissue oxygenation index; wHVx, wavelet hemoglobin volume index; wPRx, wavelet‐based pressure reactivity index.

#### 3.2.2 MAPopt versus MAPopt (Table 2)

Nine studies compared MAPopt (also referred to as ABPopt) derived from different autoregulatory indices across heterogeneous patient populations, including cardiac surgery, aSAH, neonatal HIE, TBI, and generalized critical illness. Among the included studies, four studies had fewer than 20 subjects.

In cardiac surgery cohorts, studies consistently reported strong agreement between TCD‐ and NIRS‐based indices. Blaine Easley et al. (2013) and Hori et al. (2015) found mean MAPopt values of ~74 mmHg across both modalities, with correlations of *r* = 0.59–0.71 and biases ≤3 mmHg. Liu et al. (2022) corroborated these findings, showing nearly identical MAPopt from Mx (TCD‐based) and COx_a (NIRS‐based) (75.5 vs. 76.8 mmHg).

In neurocritical care settings, strong correlations were also observed. Rivera‐Lara et al. (2017) demonstrated high agreement between COx_a‐ and Mx‐derived MAPopt in comatose patients (*R* = 0.87), while Silverman et al. (2019) reported excellent agreement between NIRS‐ and ICP‐derived MAPopt (autoregulatory indices: TOx (tissue oxygenation index) by NIRS and PRx by ICP) in aSAH (*p* = 0.93, bias: −0.7 mmHg). Bindra et al. (2016) in contrast found only a moderate correlation (*r* = 0.47, *p* = 0.003) between invasive and entirely noninvasive NIRS‐derived ABPopt, with wide limits of agreement. Oshorov et al. (2022) found moderate correlation between PRx and COx_a derived MAPopt (*r* = 0.49, *p* < 0.038) with good agreement of bias (0.39 ± 7.89). Additionally, Hoiland et al. (2020) observed wider agreement limits (±25 mmHg) between PRx‐ and COx_a‐derived MAPopt in post‐cardiac arrest patients, underscoring the variability across contexts.

Overall, MAPopt/ABPopt estimates across Table 2 demonstrate a strong correlation, with NIRS‐based metrics offering a feasible non‐invasive alternative to invasive ICP‐based measures.

#### 3.2.3 BISopt versus BISopt (Table 3)

Only one study directly compared BISopt derived from different autoregulatory indices. In a TBI cohort, Froese, Gomez, Sainbhi, Batson, Slack, et al. (2022) found no significant difference between BISopt derived from COx_a and PRx (median values 48 vs. 45, *p* = 0.31). This suggests that BISopt can be derived robustly from multiple indices, though evidence remains sparse. This study recruited 42 TBI patients.

#### 3.2.4 Cross‐opt comparisons (Table 4)

Two studies investigated relationships between BISopt and perfusion‐related Opts. In moderate‐to‐severe TBI, Froese, Gomez, Sainbhi, Batson, Stein, et al. (2022) reported no correlation between BISopt and CPPopt (*r* = 0.15, *p* = 0.38). This study recruited 32 TBI patients. In a subsequent study including both healthy controls and surgical patients, Froese et al. (2024) found a limited relationship between BISopt and MAPopt derived from COx_a. These results suggest that BISopt may represent a distinct physiologic optimization target, independent of perfusion‐related Opts. This study recruited 57 healthy controls and 27 elective surgery patients. However, the comparison between BISopt and CPPopt may be too complex to be fully captured by simple correlation coefficients. From a physiologic perspective, it may be more informative to evaluate whether the CPP values present at BISopt correlate with CPPopt, as sedative agents commonly used to achieve BIS targets can lower arterial pressure and consequently influence CPP (Oddo et al., 2016). Future studies should therefore consider comparing the CPP associated with BISopt to CPPopt at corresponding time periods to clarify whether these represent truly independent or interrelated optimization mechanisms.

Across CPPopt, MAPopt/ABPopt, and BISopt, multiple autoregulatory indices have demonstrated comparable estimates of optimal physiological targets. PRx remains the most validated and widely used reference standard for Opt indices, but other alternative indices such as wPRx, L‐PRx, HVx, and COx/COx_a show strong agreement and may provide better advantages in specific contexts. NIRS‐based methods show promise as a noninvasive surrogate for ICP‐based monitoring, although noninvasive approaches demonstrate greater variability. These findings support the feasibility of deriving consistent Opt values across multiple indices and reinforce the robustness of autoregulatory derived monitoring in critical care.

## 4 DISCUSSION

This systematic scoping review synthesized 21 studies comparing optimal physiologic targets: CPPopt, MAPopt, and BISopt derived from different CVR indices across a spectrum of patient populations. The overarching finding is that both CPPopt and MAPopt demonstrate strong reproducibility across invasive and noninvasive monitoring modalities, whereas BISopt appears to represent a distinct optimization domain, potentially reflecting sedation‐related neurovascular coupling physiology rather than perfusion.

### 4.1 CPPopt across indices

CPPopt was the most extensively evaluated parameter, particularly in TBI cohorts. Studies consistently demonstrated convergence of CPPopt values across ICP‐derived indices such as PRx, PAx, and RAC, with mean differences typically less than 3 mmHg and median values clustering between 70 and 76 mmHg. These findings underscore the robustness of CPPopt within ICP‐based frameworks and highlight its potential as a reliable physiologic target in neurocritical care.

Advances in signal processing further enhanced CPPopt derivation. For example, wPRx (wavelet‐based) provided improved stability and greater yield compared with the traditional PRx and the DATACAR method improved yield compared with the traditional PRx, while low‐resolution long PRx (L‐PRx) generated comparable values, though prognostic performance was weaker. When compared with noninvasive alternatives, NIRS‐derived indices such as COx and THx correlated strongly with PRx‐derived CPPopt, while brain tissue oxygenation‐based ORx and cerebral blood flow‐derived indices demonstrated weaker or absent associations. Collectively, these results suggest that while ICP‐based indices remain the reference standard, NIRS‐based methods may offer clinically feasible non‐invasive surrogates, particularly when invasive monitoring is impractical.

### 4.2 MAPopt across indices

MAPopt was investigated across diverse populations, including cardiac surgery, neonatal hypoxic–ischemic encephalopathy (HIE), aneurysmal subarachnoid hemorrhage (aSAH), TBI, and generalized critical illness. Across these settings, MAPopt values derived from TCD‐ and NIRS‐based indices (Mx, HVx, COx_a, TOx, THx) demonstrated strong correlations, often yielding nearly identical estimates around 70–76 mmHg.

In neurocritical care cohorts, NIRS‐based indices demonstrated excellent agreement with both TCD‐ and ICP‐derived MAPopt, particularly in aSAH and comatose patients. However, some variability persists: for instance, post‐cardiac arrest populations showed wide agreement limits (±20–25 mmHg) between PRx‐ and COx_a‐derived MAPopt, raising caution for bedside application in high‐risk contexts.

### 4.3 BISopt across indices and in relation to perfusion targets

In contrast to CPPopt and MAPopt, evidence for BISopt remains limited. The single study directly comparing BISopt across indices found no significant differences between values derived from PRx and NIRS‐based COx_a in TBI patients, suggesting internal consistency. However, when BISopt was compared with perfusion‐related Opts, no meaningful correlation with either CPPopt or MAPopt was observed. These findings imply that BISopt reflects a physiologic optimization process distinct from perfusion, more likely aligned with neurovascular coupling and sedation depth. These preliminary results indicate that BISopt may complement sedation strategies but should not be considered a replacement for perfusion optimization.

From a practical standpoint, several commercially available platforms now provide automated or semi‐automated calculation of cerebrovascular reactivity (CVR) indices and derived “optimal” parameters, such as CPPopt. For example, the ICM+ software (Cambridge Enterprise, UK) remains the most widely used research tool, enabling real‐time computation of PRx and continuous fitting of CPP‐PRx relationships to estimate CPPopt using validated algorithms (Smielewski et al., 2024). Similarly, Moberg Analytics (Natus, USA) integrates multimodal neuromonitoring data, including ICP, ABP, and EEG, and offers modules for autoregulation tracking within its Moberg CNS Monitor system (Moberg Analytics, 2025). Even though these systems illustrate the increasing translation of autoregulation monitoring from research software to clinically integrated tools, they still suffer from inter‐platform variability and lack of transparency regarding Opt parameter calculation methodology (Beqiri et al., 2023; Smielewski et al., 2024; Stein et al., 2025).

### 4.4 Clinical implications

Taken together, this body of work highlights the potential of noninvasive, autoregulation‐derived Opt parameters to serve as proxy tools for invasive Opt management. CPPopt and MAPopt demonstrate physiologic robustness across both invasive and noninvasive modalities, supporting their potential interchangeability within multimodal monitoring frameworks.

Recent evidence from the secondary analysis of the COGiTATE randomized controlled trial demonstrated that, within the intervention arm of the trial, PRx was closer to PRxopt (PRx value at CPPopt is PRxopt) when PRxopt was more negative during preserved autoregulation (Beqiri et al., 2025). Although no overall difference in mean PRx was observed between the intervention and control groups in the main trial, this finer‐grained analysis supports the physiological rationale that CPPopt targeting can enhance autoregulatory function when a margin for improvement exists (Beqiri et al., 2025). Additionally, many studies found that deviation of parameters such as MAP or CPP from the optimal state is associated with worse outcomes, which emphasizes the importance of adopting these optimal parameter‐based treatment plans in neurocritical care for better outcomes in the future (Xie et al., 2025). The clinical integration of these parameters will depend on methodological standardization, prospective outcome‐driven validation, and development of bedside‐compatible analytic tools. As precision medicine advances in neurocritical care and perioperative management, individualized Opt targets may serve as critical anchors for personalized therapies.

## 5 LIMITATIONS AND CHALLENGES

This review has several important limitations that should be acknowledged. First, the heterogeneity of included studies is substantial. Populations ranged from adults with severe TBI to neonates with hypoxic–ischemic encephalopathy and patients undergoing cardiac surgery or recovering from cardiac arrest. This diversity reflects the broad applicability of autoregulation‐derived targets, but it also complicates direct comparison across settings. Second, methodological variability was considerable. Studies differed in the autoregulatory indices applied (e.g., PRx, PAx, RAC, COx, COx_a, HVx, THx, wPRx, and wHVx), the physiologic signals used (ICP, NIRS, TCD, brain tissue oxygenation, skeletal muscle oxygenation), and the analytic approaches (window length, curve‐fitting techniques, and binning strategies). While some refinements, such as wavelet‐based methods, improved yield and stability, they also introduced analytic complexity. The lack of standardized methodology limits cross‐study comparability and hinders clinical translation. Third, although agreement across indices was generally strong, wide limits of agreement were reported in several studies (e.g., ±20–25 mmHg between PRx‐ and COx‐derived MAPopt). This degree of variability raises concerns about clinical interchangeability, particularly in scenarios requiring precise hemodynamic control. Fourth, the validity of CVR indices can deteriorate due to the presence of artifacts in biosignals such as intracranial pressure and arterial blood pressure (Rozanek et al., 2022). Proper removal of artifacts can be a real concern to establish the validity of the results of a study. Fifth, the small sample sizes in some studies limit statistical power and heighten the likelihood of type II errors. Finally, BISopt research remains very limited. With only three published studies: one comparing BISopt across indices and two examining its relationship to CPPopt/MAPopt, current evidence is insufficient to define its role in critical care or anesthesia.

## 6 FUTURE DIRECTIONS

Future work in this field should focus on addressing several key gaps. A major priority is the development of standardized methodologies for Opt derivation, including agreement on data window lengths, curve‐fitting approaches, binning strategies, and yield reporting. The current heterogeneity in analytic techniques makes cross‐study comparisons difficult and hampers clinical adoption. In parallel, there is a pressing need for outcome‐driven validation. While many studies have demonstrated feasibility and correlation between indices, far fewer have examined whether targeting CPPopt, MAPopt, or BISopt improves survival, neurological recovery, or other patient‐centered outcomes. Prospective interventional trials in TBI, aSAH, cardiac arrest, and perioperative settings will be critical to establish the true therapeutic value of these parameters.

Another important avenue is the further exploration of BISopt. The limited existing studies suggest that BISopt reflects a domain distinct from perfusion‐related optimization, potentially aligning with sedation depth rather than hemodynamic targets. Future research should clarify whether BISopt‐guided sedation strategies enhance autoregulatory stability or functional recovery, both in neurocritical care and in perioperative contexts. At the same time, multimodal integration of ICP, NIRS, TCD, EEG, and brain tissue oxygenation could strengthen the reliability of Opt derivation by enabling cross‐validation across signals and providing clinicians with convergent targets. Finally, the translation of Opt monitoring into noninvasive and even non‐cerebral applications represents an exciting frontier. Evidence from NIRS‐based indices and recent work in skeletal muscle autoregulation suggests that individualized optimization may extend beyond cerebral physiology, opening the door to wider applicability in pediatric, perioperative, and general critical care populations where invasive monitoring is not feasible.

## 7 CONCLUSION

This scoping review demonstrates that CPPopt and MAPopt can be derived consistently across a variety of autoregulatory indices, with both invasive and noninvasive modalities yielding broadly comparable estimates. These findings underscore the physiological robustness of autoregulation‐derived perfusion targets, though methodological variability and wide limits of agreement continue to pose challenges for clinical implementation. In contrast, BISopt appears to capture a distinct optimization domain related more to sedation depth than to perfusion, offering a potentially complementary tool in individualized patient management. Despite the encouraging physiological consistency demonstrated across studies, translation into clinical practice remains limited by heterogeneity in derivation methods and a lack of outcome‐based validation. The next steps must therefore include methodological standardization, multimodal integration of monitoring techniques, and rigorous prospective trials that directly test whether Opt‐guided care improves neurological and systemic outcomes. If these challenges can be addressed, CPPopt, MAPopt, and BISopt have the potential to move beyond experimental constructs and become essential components of precision medicine in critical care, offering clinicians actionable, individualized targets to guide both perfusion and sedation management.

## FUNDING INFORMATION

This work was directly supported through the Endowed Manitoba Public Insurance (MPI) Chair in Neuroscience and the Natural Sciences and Engineering Research Council of Canada Alliance Advantage program (NSERC; ALLRP‐597708‐24; Medtronic ERP‐2024‐14025).

## CONFLICT OF INTEREST STATEMENT

F.A.Z. currently has NSERC Alliance Advantage grant (ALLRP‐597708‐24) support in partnership with Medtronic's Acute Care & Monitoring Division (ERP‐2024‐14025) for work that is related to this manuscript. Funding from the partner organization is provided to match NSERC governmental funding only, in keeping with NSERC policies. Medtronic does not direct the research objectives, data collection, analysis, interpretation, or publication of the findings in any way. All other authors assert that they have no conflicts of interest regarding this work, confirming the absence of any financial interests, affiliations, or personal relationships that may have influenced or biased this research.

## Supporting information


Table S1.

## Appendix

The detailed strings of keywords that were used as search parameters in BIOSIS, SCOPUS, EMBASE, MEDLINE, Global Health, and Cochrane Library were as follows:

“MAPopt” OR “Mean Arterial Pressure optimization” OR “MAP opt” OR

“Mean arterial pressure optimal” OR “Mean arterial pressure optimum”

OR “Optimal mean arterial pressure” OR “Optimum mean arterial pressure”

“Blood pressure optimal” OR

“Blood pressure optimum” OR “MAP optimization” OR

“ABP opt” OR “ABPopt” OR “Arterial blood pressure optimum” OR

“Optimum arterial blood pressure” OR “arterial blood pressure optimal”

OR “Optimal arterial blood pressure” OR

“Arterial blood pressure optimization” OR “CPPopt” OR

“Cerebral Perfusion Pressure optimum” OR “Optimum Cerebral Perfusion Pressure”

OR “Optimal CPP” OR “Optimal Cerebral Perfusion Pressure” OR

“Cerebral Perfusion Pressure optimal” OR “cerebral perfusion pressure optimization”

OR “CPP optimization” OR “Individualized CPP” OR “targeted cerebral perfusion pressure”

OR “cerebral autoregulation‐guided CPP” OR “personalized cerebral perfusion pressure”

OR “optimized brain perfusion pressure” OR “optimal perfusion pressure” OR

“real‐time CPP optimization” OR “BISopt” OR “Optimal BIS” OR

“Bispectral Index optimization” OR “BIS optimization”

OR “optimal Bispectral Index level” OR “target BIS” OR “individualized BIS”

OR “BIS‐guided anesthesia” OR “depth of anesthesia targeting” OR

“BIS titration” OR “optimal sedation depth” OR “personalized sedation target”

OR “BIS‐based sedation management” OR “tailored anesthesia depth”
