# Supplementary material for: Intercerebral autoregulation index consistency in the derivation of CPPopt, MAPopt, and BISopt in humans: A scoping review
Source: Physiol Rep. 2025 Dec 19;13(24):e70660. doi: 10.14814/phy2.70660 (PMC12717444; doi:10.14814/phy2.70660)
Supplement: Supplementary file 1 — Table S1. [file PHY2-13-e70660-s001.docx]

**Table S1. PRISMA ScR Checklist**

| **Section** | **Item** | **PRISMA-ScR Checklist Item** |
| --- | --- | --- |
| **Title** | 1 | See pg. 1 |
| **Abstract** |  |  |
| Structured summary | 2 | See pg. 5 (Abstract) |
| **Introduction** |  |  |
| Rationale | 3 | See pg. 5-6 (Section 1) |
| Objectives | 4 | See pg. 5-6 (Section 1) |
| **Methods** |  |  |
| Protocol and registration | 5 | N/A |
| Eligibility criteria | 6 | See pg. 7 (Section 2.2) |
| Information sources | 7 | See pg. 7 (Section 2.3) |
| Search | 8 | See pg. 7 (Section 2.3) |
| Selection of sources of evidence | 9 | See pg. 7 (Section 2.4) |
| Data charting process | 10 | All relevant data were collected from each article by the primary author |
| Data items | 11 | See pg. 7 (Section 2.5) |
| Critical appraisal of individual sources of evidence | 12 | N/A |
| Summary measures | 13 | N/A |
| Synthesis of results | 14 | All data items for each method were tabulated and are included in Tables 1-5 |
| Risk of bias across studies | 15 | As such, all articles published in academic journals were assumed to have been screened. |
| Additional analyses | 16 | N/A |
| **Results** |  |  |
| Selection of sources of evidence | 17 | See pg. 8-38 (Section 3) |
| Characteristics of sources of evidence | 18 | See Tables 1-5 |
| Critical appraisal of sources of evidence | 19 | N/A |
| Results of individual sources of evidence | 20 | See Tables 1-5 |
| Synthesis of results | 21 | All articles included a valid relationship between Opt parameters such as CPPopt, MAPopt and BISopt. |
| Risk of bias across studies | 22 | As such, all articles published in academic journals were assumed to have been screened. |
| Additional analysis | 23 | N/A |
| **Discussion** |  |  |
| Summary of evidence | 24 | See pg. 38-39 (Section 4) |
| Limitations | 25 | See pg. 40 (Section 5) |
| Conclusions | 26 | See pg. 41 (Section 7) |
| **Funding** | 27 | See pg. 3-4 (Funding) |
